# Associations Between Adverse Childhood Experiences and Physical Activity, Recreational Screen Time, and Sleep Among U.S. Children

**DOI:** 10.3390/bs16040598

**Published:** 2026-04-17

**Authors:** Eunice Lee

**Affiliations:** School of Social Work, College of Health, Cleveland State University, 2121 Euclid Avenue, Rhodes Tower #1412, Cleveland, OH 44115, USA; e.lee85@csuohio.edu

**Keywords:** adverse childhood experiences, screen time, sleep, physical activity, health behaviors

## Abstract

Adverse childhood experiences (ACEs) are a public health concern in the United States. Using the 2019 National Survey of Children’s Health, this cross-sectional secondary analysis examined associations between cumulative ACEs (0, 1, 2, and 3 or more) and three health behaviors among children ages 6 to 17, including physical activity, recreational screen time, and sleep. Interaction models were also estimated by child sex and race/ethnicity (White non-Hispanic, Black non-Hispanic, and Hispanic) to assess whether these associations differed across groups. Nearly half of children experienced at least one ACE, and about one in eight experienced three or more. In adjusted models, higher numbers of ACEs were associated with a lower likelihood of meeting recreational screen time guidelines and sleep recommendations, while no statistically significant association was observed for meeting physical activity recommendations. Interaction analyses by child sex and race/ethnicity found no statistically significant differences in these associations across groups. These findings suggest that children with higher numbers of ACEs may be less likely to meet recommended sleep and recreational screen time guidelines, underscoring the potential value of trauma-informed strategies that strengthen sleep routines and healthy media practices.

## 1. Introduction

Adverse childhood experiences (ACEs) refer to stressful and potentially traumatizing life events occurring before the age of 18. These experiences can include child maltreatment, household challenges, exposure to violence or crime, or economic hardship (Felitti et al., 1998; Giano et al., 2020). ACEs represent a substantial public health concern in the United States. In 2022, national estimates indicate that 39.7% of U.S. children had experienced at least one ACE, and 17.8% had experienced two or more (HRSA Maternal and Child Health Bureau, 2026). ACEs are also influenced by social and structural conditions. Children and families living in economically disadvantaged neighborhoods and children from racial and ethnic minority families are more likely to experience higher numbers of ACEs, reflecting broader inequities that influence exposure to stressors (Giano et al., 2020).

Since the seminal ACE study by Felitti et al. (1998), extensive research has documented associations between ACEs and a wide range of health outcomes across the course of life. ACEs have been linked to depression (Chapman et al., 2004; Elmore & Crouch, 2020; Leban & Gibson, 2020; Lee et al., 2020), substance use disorders (Leban & Gibson, 2020; Shin et al., 2018), suicidal behaviors (Dube et al., 2001; Thompson et al., 2019), increased healthcare costs (Koball et al., 2019; Miller et al., 2020), and chronic diseases such as obesity (Elsenburg et al., 2017; Wiss & Brewerton, 2020), cardiovascular disease (Godoy et al., 2021; Su et al., 2015; Suglia et al., 2018), and cancer (Holman et al., 2016). Importantly, prior research consistently supports a dose–response relationship, such that the likelihood of negative outcomes increases as the number of ACEs rises (Bethell et al., 2017; Felitti et al., 1998; Garner et al., 2021; Hughes et al., 2017; Jones et al., 2020; Swedo et al., 2024). For example, Felitti et al. (1998) documented a graded pattern in which higher cumulative ACE counts were associated with greater risk of multiple adult health risk factors and leading causes of mortality. A systematic review and meta-analysis similarly found that individuals reporting four or more ACEs experienced substantially elevated risk across a broad range of outcomes compared with those reporting none (Hughes et al., 2017). Using Youth Risk Behavior Survey data, Swedo et al. (2024) also found particularly strong associations between four or more ACEs and suicidal ideation, suicide attempts, and prescription opioid misuse.

A growing body of research also links ACEs to children’s health-related behaviors, including physical activity, screen time, and sleep. Prior studies suggest that a higher number of ACEs may be associated with lower levels of physical activity. For example, Raney et al. (2022) found that higher cumulative ACE scores were associated with fewer hours of physical activity and fewer days meeting the 60 min per day benchmark among youth. More recent work using objective indicators such as daily steps similarly reported an inverse graded relationship in which higher cumulative ACEs were associated with fewer daily steps (Al-shoaibi et al., 2024). Prior research has also documented associations between cumulative ACEs and screen-based behaviors. Studies using nationally representative data suggest that higher numbers of ACEs are associated with greater digital media use and more recreational screen time (Jackson et al., 2021; Raney et al., 2022). A substantial literature also links cumulative ACEs to poorer sleep health, including shorter sleep duration and more sleep problems (Desch et al., 2023; Hunt et al., 2024; Lin et al., 2022; Qu et al., 2024; Rojo-Wissar et al., 2021). These patterns are biologically plausible given evidence that chronic stress may dysregulate stress response systems, increase physiological arousal, and disrupt circadian rhythms in ways that can interfere with sleep onset and continuity (Buckley & Schatzberg, 2005; Koss & Gunnar, 2018).

Given the serious consequences associated with cumulative ACEs, it is important to identify modifiable behaviors that may be linked to health outcomes among children with a history of childhood adversity (Su et al., 2015; Suglia et al., 2018). Physical activity, recreational screen time, and sleep are particularly relevant because they are embedded in daily routines and amenable to prevention and intervention strategies. However, limited research has examined these associations in nationally representative samples of children while also accounting for multilevel factors that influence activity, screen time, and sleep, including child characteristics, family socioeconomic conditions, and neighborhood and contextual environments. Further, because cumulative ACEs and health behaviors may vary across subgroups, it is important to examine whether these associations are consistent across groups and whether tailored trauma-informed prevention and intervention strategies should be considered.

To address these gaps, this study examined associations between cumulative ACEs categorized as 0, 1, 2, and 3 or more, and three modifiable health behaviors among children ages 6 to 17. The behaviors were meeting physical activity recommendations (at least 60 min per day), meeting recreational screen time guidelines (2 h or less per day), and meeting age-specific sleep guidelines (9–11 h per night for children ages 6 to 12 and 8 to 10 h per night for adolescents ages 13 to 17). The study also explored whether these associations differed by child sex and race/ethnicity, including White non-Hispanic, Black non-Hispanic, and Hispanic children. It was hypothesized that a higher number of ACEs would be associated with a lower likelihood of meeting physical activity recommendations, recreational screen time guidelines, and sleep guidelines. It was also hypothesized that these associations might differ by child sex and race/ethnicity.

## 2. Methods

### 2.1. Data Source and Sample

**Figure 1:**
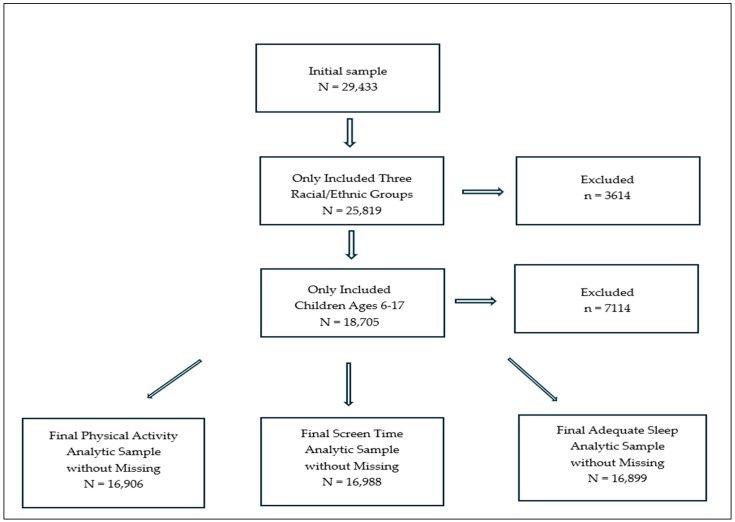
Final Analytic Sample Selection Flow Diagram.

This study utilized data from the 2019 National Survey of Children’s Health (NSCH), a cross-sectional survey sponsored by the Health Resources and Services Administration (HRSA)’s Maternal and Child Health Bureau (MCHB). The data were weighted to represent the entire population of non-institutionalized U.S. children between less than 1 year and 17 years of age at the time of sampling (HRSA Maternal and Child Health Bureau, 2020). The survey respondents were parents or guardians of a randomly selected child within their household (hereafter referred to as “parents”). The NSCH gathered information on children’s health and well-being, along with factors related to their families and communities. The survey was administered in both English and Spanish. The overall response rate for the 2019 survey was 42.4%. For the 2019 NSCH, a total of 68,500 initial screener surveys were completed between June 2019 and January 2020, with 29,433 households completing a topical interview (HRSA Maternal and Child Health Bureau, 2020). As shown in Figure 1, the analytic sample was limited to children classified as White non-Hispanic, Black non-Hispanic, or Hispanic, and to those ages 6 to 17 years, yielding an eligible sample of 18,705 children. Analytic samples were then defined separately for each outcome using complete case analysis, also referred to as listwise deletion, which included only children with non-missing values on the outcome, cumulative ACEs, and all covariates in the corresponding model. The final analytic sample sizes were 16,906 for physical activity, 16,988 for limited screen time, and 16,899 for adequate sleep. Missing data diagnostics were conducted using binary indicators of missingness to assess whether missingness was related to cumulative ACEs and each outcome variable. These analyses indicated that missingness was not completely random. Accordingly, although complete-case analysis offers a transparent and reproducible approach, it can reduce precision and may introduce bias when missingness is related to the outcome or other variables in the analysis, or to unmeasured factors associated with these variables (White & Carlin, 2010). This study received exempt status from the Institutional Review Board of [Name Redacted] University.

### 2.2. Measures

#### 2.2.1. Outcome Variables: Physical Activity, Limited Screen Time, and Adequate Sleep

Physical activity was measured in the NSCH by the number of days a child engaged in physical activities, such as exercise or sports, for at least 60 min per day. Parents were asked, “During the past week, on how many days did this child exercise, play a sport, or participate in physical activity for at least 60 min?” This variable had four response options, ranging from “0 days” to “every day.” Based on CDC guidelines recommending at least 60 min of moderate- to vigorous-intensity physical activity per day for children aged 6 and 17 years (U.S. Department of Health and Human Services, 2018), children were categorized as “yes” (every day) or “no” (0–6 days) for the analysis.

Screen time was defined as time spent watching television, playing games, or using computers, cell phones, or other electronic devices for recreational purposes. Parents were asked, “On most weekdays, about how much time did this child spend in front of a TV, computer, cellphone, or other electronic device watching programs, playing games, accessing the internet, or using social media?” Response options included: less than 1 h, 1 h, 2 h, 3 h, and 4 or more hours. Consistent with prior studies defining appropriate hours of screen exposure (Guram & Heinz, 2018), children were categorized as “yes” (≤2 h) or “no” (≥3 h) for the analysis.

The NSCH assessed average sleep duration by asking parents, “During the past week, how many hours of sleep did this child get on most weeknights?” The initial response options ranged from less than 6 h to 11 or more hours. These responses were collapsed into a binary variable (yes/no), indicating whether the child met the American Academy of Pediatrics (AAP) sleep recommendations. The AAP recommends 9–11 h of sleep per night for children aged 6–12 years and 8–10 h per night for adolescents aged 13–17 years (Paruthi et al., 2016).

#### 2.2.2. Predictor: Adverse Childhood Experiences (ACEs)

The NSCH measured ACEs using nine parent-reported items that were adapted from the original CDC Kaiser ACE study. These items were developed with input from a technical expert panel and were evaluated through cognitive testing by the CDC’s National Center for Health Statistics (HRSA Maternal and Child Health Bureau, 2020). Importantly, the NSCH ACE module does not include abuse and neglect items included in the original CDC Kaiser ACE study, including physical, emotional, and sexual abuse and physical and emotional neglect. Therefore, cumulative ACEs in this study reflect an NSCH-specific set of adversities. Specifically, parents were asked, “To the best of your knowledge, has this child ever experienced any of the following?” The nine listed childhood adversities were:

1. Difficulty covering basic needs like food or housing;
2. Parent or guardian divorce or separation;
3. Death of a parent or guardian;
4. Parent or guardian incarceration;
5. Witnessing physical violence between adults in the home;
6. Being a victim of or witnessing violence in the neighborhood;
7. Living with someone who is mentally ill, suicidal, or severely depressed;
8. Living with someone who has alcohol or drug problems;
9. Being treated or judged unfairly based on race or ethnicity.

Each ACE item was coded as “yes” or “no” except for economic hardship. Economic hardship was initially assessed using a 4-point Likert scale ranging from “never” to “very often” and then dichotomized as “yes” (somewhat often/very often) or “no” (rarely/never). The cumulative ACE score was created by summing items, producing values from 0 to 9. For analysis, cumulative ACEs were categorized into four groups (0, 1, 2, and 3 or more ACEs), with 0 ACEs serving as the reference group.

#### 2.2.3. Covariates

Covariates were grouped into child-, parent-, household-, and neighborhood-level factors. Child-level factors included sex, age in years, race/ethnicity, and special health care needs (CSHCN). For stratified analyses by race and ethnicity, the analytic sample was limited to White non-Hispanic, Black non-Hispanic, and Hispanic children. The NSCH identified children with special health care needs using five screening questions related to prescription medication use, functional limitations, medical or mental health services, specialized therapies, and treatment for emotional or developmental problems (Bethell et al., 2002). If a parent responded affirmatively to any of these questions, and the condition was expected to last at least 12 months, the child was classified as having special health care needs. Parent- and household-level factors included the mother’s age, parental educational attainment, family structure, household size, household income as a percentage of the federal poverty level, and participation in government assistance programs such as food or cash assistance within the past year.

Neighborhood factors were assessed using three measures. Specifically, supportive neighborhood was measured with three parent-reported items: (1) people in the neighborhood help each other out, (2) neighbors watch out for each other’s children, and (3) families know where to go for help in the community. A neighborhood was classified as supportive if the parent “definitely agreed” with at least one item and “somewhat agreed” or “definitely agreed” with the other two items. Neighborhood amenities were assessed by counting the number of four specific amenities present in the neighborhood: (1) sidewalks or walking paths, (2) parks or playgrounds, (3) recreation centers, community centers, or boys’ and girls’ clubs, and (4) libraries or bookmobiles. Each item was coded as “1” if present and “0” otherwise. A binary variable was then constructed to indicate the presence of neighborhood amenities (1 = all four amenities present, 0 = otherwise). Distracting neighborhood conditions were assessed by counting the number of three detracting factors present: (1) litter or garbage on the street or sidewalk, (2) poorly maintained or rundown housing, and (3) vandalism, such as broken windows or graffiti. Each item was coded as “1” if present and “0” otherwise. A binary variable was then constructed to indicate distracting neighborhood conditions (1 = all three detracting factors present; 0 = otherwise).

### 2.3. Statistical Analyses

Both unweighted and weighted frequency distributions were examined for all study variables. Associations between cumulative ACEs and the three modifiable health outcomes (physical activity, limited screen time, and adequate sleep) were examined using multivariable logistic regression models that adjusted for child, parent, household, and neighborhood covariates. Interaction terms between cumulative ACEs and child sex and between cumulative ACEs and race/ethnicity were then estimated to assess whether these associations differed across groups. Due to the substantial amount of missing data for household income, six imputed federal poverty level variables provided by the MCHB were included in the analyses using the multiple imputation function (i.e., mi estimate). All analyses were conducted in STATA (version 18). To account for the complex survey design, cluster and sampling weights were applied to adjust for nonresponse and unequal selection bias. Statistical significance was set at *p* < 0.05.

## 3. Results

### 3.1. Sample Characteristics

Descriptive characteristics of the study sample are shown in Table 1. The sample included slightly more males (51.5%), with an average child age of 11.6 years (SD = 3.4). More than half of children were White (55.9%), followed by Hispanic (28.6%) and Black (15.5%). About 23.6% of children were classified as having special health care needs. The average age of mothers was 29 years (SD = 6.1), and 11.1% of parents did not have a high school degree. Most children (69.9%) lived with two parents. Approximately 41% of households lived below 200% of the federal poverty level, and 39.2% received food or cash assistance within the past 12 months. The average household size was 4.4 people (SD = 1.3). In terms of neighborhood characteristics, 55.1% of children lived in a supportive neighborhood, 86.7% lived in neighborhoods with amenities, and 27.4% lived in neighborhoods with distracting conditions.

### 3.2. Prevalence of Physical Activity, Limited Screen Time, and Adequate Sleep

**Figure 2:**
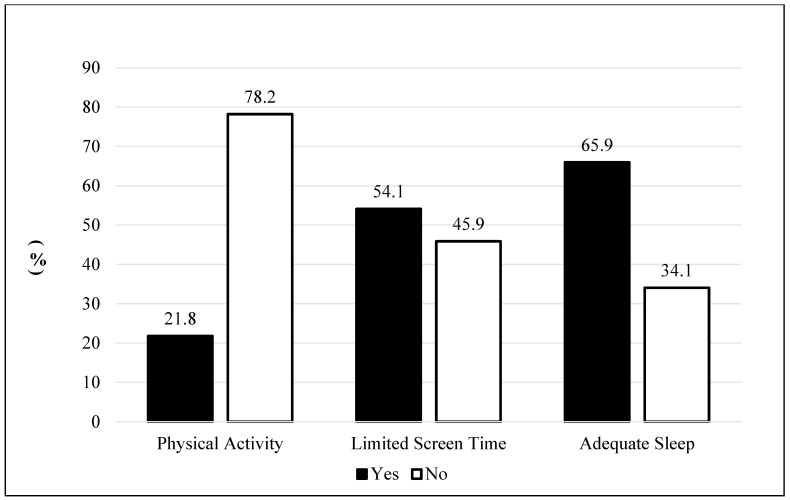
Prevalence of Physical Activity, Limited Screen Time, and Adequate Sleep. *Notes.* Weighted proportions are presented. Outcome definitions: Physical activity (=Yes) indicates 60 min of activity every day in the past week; Limited screen time (=Yes) indicates ≤2 h of recreational screen time on most weekdays; Adequate sleep (=Yes) indicates 9–11 h/night for ages 6–12 and 8–10 h/night for ages 13–17.

Figure 2 shows the prevalence of physical activity, limited screen time, and adequate sleep in the sample. About 21.8% of children engaged in at least 60 min of physical activity every day and 54.1% had 2 h or less of screen time on most weekdays. In addition, 65.9% met the recommended sleep guidelines.

### 3.3. Prevalence of ACEs

Table 2 presents descriptive characteristics of individual and cumulative ACEs in the sample. Slightly less than half of children (45.4%) experienced at least one ACE, with 12.8% reporting three or more ACEs. The most common ACE was parental divorce or separation (29.5%), whereas parental death was the least common (4.1%).

### 3.4. Associations Between ACEs and Physical Activity, Limited Screen Time, and Adequate Sleep

Table 3 presents the results of the multivariate logistic regression models. After adjusting for individual-, parent-, household-, and neighborhood-level covariates, ACEs showed significant negative associations with limited screen time and adequate sleep. Specifically, children with 1 ACE and 2 ACEs had 26% (AOR = 0.74, 95% CI = 0.61, 0.89, *p* < 0.01) and 30% (AOR = 0.70, 95% CI = 0.53, 0.92, *p* < 0.05) lower odds of having limited screen time, respectively, compared with children with 0 ACEs. Children with 3 or more ACEs also had 31% (AOR = 0.69, 95% CI =0.51, 0.93, *p* < 0.05) lower odds of having limited screen time than those with 0 ACEs. For sleep, children with 2 ACEs had 30% (AOR = 0.70, 95% CI = 0.52, 0.92, *p* < 0.05) lower odds of having age-appropriate adequate sleep compared with children with 0 ACEs. Additionally, children with 3 or more ACEs had 33% (AOR = 0.67, 95% CI = 0.50, 0.89, *p* < 0.01) lower odds of having adequate sleep than their counterparts with 0 ACEs. No significant associations were found between ACEs and physical activity.

### 3.5. Interactions Effects Between ACEs and Physical Activity, Limited Screen Time, and Adequate Sleep by Child Sex and Race/Ethnicity

Table 4 presents the interaction effects of ACEs with child sex and race/ethnicity on physical activity, limited screen time, and adequate sleep. These interaction models were adjusted for the same individual, parent, household, and neighborhood covariates included in the main models. For physical activity, the interaction between Black race/ethnicity and 1 ACE was significant (AOR = 1.78, 95% CI =1.02, 3.10, *p* < 0.05), suggesting that the association of 1 ACE with physical activity differed for Black children relative to White non-Hispanic children. For adequate sleep, the interaction between Hispanic ethnicity and 2–3 ACEs was significant (AOR = 0.46, 95% CI =0.24, 0.87, *p* < 0.05), indicating a stronger negative association between 2 and 3 ACEs and adequate sleep among Hispanic children relative to White non-Hispanic children. However, the omnibus interaction tests indicate that the associations between ACEs and these three health behaviors did not differ significantly by child sex or race/ethnicity.

## 4. Discussion

### 4.1. Associations Between ACEs and Three Health Behaviors

This study examined associations between cumulative ACEs and three health behaviors, including physical activity, recreational screen time, and sleep, and assessed whether these associations varied by child sex and race/ethnicity. Using a nationally representative sample of U.S. children ages 6 to 17, the study documents both the high prevalence of ACEs and their associations with health behaviors. Specifically, nearly half of children experienced at least one ACE, and about one in eight experienced three or more ACEs. This level of prevalence underscores that childhood adversity remains a substantial public health issue and highlights the importance of prevention strategies and policies that reduce exposure to adversity and support families in maintaining healthy daily routines. As documented in prior research (Bhutta et al., 2023; Felitti et al., 1998; Shonkoff et al., 2012), ACE-related toxic stress may be linked to disruptions in neurobiological, physiological, and emotional development, which may be associated with differences in children’s daily routines and coping behaviors and longer-term risks for mental and physical health problems.

The findings indicate that higher numbers of cumulative ACEs were associated with a lower likelihood of meeting recreational screen time guidelines. This aligns with earlier studies linking childhood adversity to greater screen engagement (Fakhouri et al., 2013; Hardy et al., 2010; Jackson et al., 2021; Raney et al., 2022; Xu et al., 2024). For example, Xu et al. (2024) found that adolescents with three or more ACEs had higher odds of excessive recreational screen use across multiple media types, even after adjusting for sociodemographic factors. One plausible interpretation is that screen use may function as an accessible coping strategy for stress or dysregulation, particularly when supportive resources are limited. This association warrants attention because excessive recreational screen time can displace sleep and physical activity and is associated with poorer mental health and higher cardiometabolic risk (Nakshine et al., 2022).

The study also found that higher numbers of cumulative ACEs were associated with a lower likelihood of meeting age-specific sleep recommendations. This is consistent with literature linking adversity to shorter sleep duration and sleep problems (Desch et al., 2023; Hunt et al., 2024; Lin et al., 2022; Qu et al., 2024; Rojo-Wissar et al., 2021). Adversity-related stress is associated with sleep disruption through pathways such as heightened physiological arousal, worry or anxiety, and dysregulated stress physiology, which can interfere with sleep initiation and sleep continuity. Prior research has shown that exposure to violence and trauma is associated with poorer sleep in children and adolescents (Kliewer & Lepore, 2015; Spilsbury et al., 2014), and trauma-related symptoms such as PTSD commonly co-occur with sleep disturbance (Kovachy et al., 2013). Evidence from specific trauma contexts also supports this link, including elevated sleep problems among adolescents exposed to childhood sexual abuse and co-occurring mental health symptoms such as depression and PTSD (Brown et al., 2022; Steine et al., 2012).

In contrast, no statistically significant association was observed between cumulative ACEs and the physical activity outcome in this study. Several factors may help explain this finding. First, the NSCH physical activity item captures daily attainment of 60 min rather than overall activity volume, intensity, or objective movement. In addition, physical activity is highly constrained by opportunity structures such as safe spaces, caregiver time, transportation, and access to programs. These constraints may reduce detectable differences across cumulative ACE categories when many children face barriers regardless of adversity history. Even without a statistically significant association in this study, physical activity remains a potentially important health behavior for children experiencing adversity because it is linked to mental and physical health benefits and may support stress management and emotional regulation (Carcelén-Fraile et al., 2025; Wang et al., 2022).

Additionally, interaction analyses were conducted to examine whether the associations between cumulative ACEs and the three health behaviors differed by child sex and race/ethnicity. Overall, the interaction effects were not statistically significant, suggesting that the associations between cumulative ACEs and three health behaviors were generally similar across child sex and race/ethnicity in this sample. This difference from prior research may reflect several factors. First, subgroup variation may depend on developmental stage, and the present study included a broad age range of children and adolescents, potentially obscuring differences that are more pronounced at particular ages. Second, variation across studies in how ACEs and health behaviors are measured, including reliance on parent-reported indicators and broad behavioral categories, may contribute to differing findings. Third, the inclusion of a broad set of child, household, and neighborhood covariates may have attenuated subgroup differences by accounting for contextual factors that are unevenly distributed across groups.

### 4.2. Practice and Policy Implications

The findings have several implications for social work practice and policy in healthcare, school, and community settings. Because higher cumulative ACEs were associated with a lower likelihood of meeting sleep recommendations and recreational screen time guidelines, these behaviors may represent particularly actionable targets for trauma-informed prevention and early intervention. The results support the value of integrating trauma-informed approaches with guidance on sleep hygiene and family media practices. In pediatric primary care and school-based health settings, clinicians and social workers may incorporate brief questions about sleep and recreational screen time into routine visits and connect families to supportive resources when they report adversity-related stressors. Such efforts may help identify children whose daily routines are being disrupted by stress before those patterns become more deeply entrenched (Strait & Meagher, 2019).

The findings also suggest the importance of family-centered approaches. Sleep and recreational screen use are often shaped by household routines, caregiver stress, and material constraints rather than solely by children’s individual choices. Accordingly, practice approaches may be most effective when focused on strengthening family routines, supporting caregiver capacity, and addressing stressors that interfere with healthy daily patterns, rather than focusing only on behavior restriction (Khalsa et al., 2024). In school settings, trauma-informed initiatives may extend beyond classroom strategies by promoting predictable routines, supportive adult relationships, and coordinated referral pathways for mental health and family support services (Avery et al., 2021). Community-based programs may also play an important role by offering structured, low-cost opportunities for after-school engagement that support healthy routines and reduce reliance on unsupervised screen time (Haycraft et al., 2020).

At the policy level, the findings underscore the importance of investments that help families maintain healthy daily routines while navigating adverse life events. Policies that expand access to affordable mental health care, including trauma-focused services for children and caregivers, may help reduce stress-related dysregulation associated with sleep disruption and increased screen use as a coping strategy. Strengthening the social safety net through stable housing support, income support, and food assistance may also promote healthier routines by reducing chronic household strain (Hock et al., 2024; Na et al., 2019). In addition, place-based investments that improve neighborhood conditions and expand access to safe green space, sidewalks, recreation facilities, and other family-supportive resources may strengthen the broader environments in which healthy routines are established and maintained. Although physical activity was not significantly associated with cumulative ACEs in the present study, it remains an important health-promoting behavior, and policies that improve access to safe and affordable opportunities for physical activity may still benefit children experiencing adversity (Reuben et al., 2020).

### 4.3. Study Limitations

While this study provides valuable insights, several limitations should be noted. First, because this study is cross-sectional, it is designed to examine associations rather than causation. The primary implication of the design is that temporal ordering cannot be established, and therefore causal pathways or precedence between cumulative ACEs and health behaviors cannot be determined. Reverse causality is possible. For example, poor sleep or high screen use could contribute to family stress and conflict, which may co-occur with or influence the reporting of adversity. Second, ACEs and health behaviors were based on parent reports, which may be subject to recall errors and social desirability bias. Because some adversities may involve caregiver or household behaviors, parents may underreport certain experiences, which could lead to underestimation of cumulative ACEs and attenuation of associations. Third, as aforementioned, the NSCH ACE module does not include abuse and neglect items captured in the original CDC Kaiser ACE framework, and the cumulative ACE measure therefore reflects an NSCH-specific set of adversities. Fourth, the physical activity and screen time measures rely on threshold-based indicators, which may be less sensitive than continuous measures or objective monitoring and could attenuate associations. Fifth, unmeasured confounding may remain despite adjustment for multiple covariates. Factors such as caregiver mental health, parenting stress, household routines, digital literacy, parental supervision practices, and aspects of the home environment such as stability and crowding could plausibly influence both cumulative ACE reporting and children’s sleep and screen time behaviors. Future research would benefit from longitudinal designs, multi-informant measurement of adversity including youth reports where developmentally appropriate, and richer measures of family routines and parental digital mediation to clarify mechanisms linking cumulative adversity with sleep and media behaviors.

## 5. Conclusions

Given the high prevalence of ACEs among children in the United States, substantial opportunities exist to strengthen multisector approaches that promote modifiable health behaviors, including recreational screen use, healthy sleep, and physical activity. Prioritizing these behaviors during childhood and adolescence may help buffer the effects of adversity on development and reduce downstream risk for mental and physical health problems. Coordinated efforts across families, schools, healthcare, and community settings—especially those that are trauma-informed and responsive to structural barriers—may support healthier daily routines and promote more positive health trajectories across the course of life.

## Figures and Tables

**Table 1:** Descriptive Characteristics of Study Sample.

Sample Characteristics (Unweighted Sample Size)	Unweighted *n* ^a^	Weighted % or Mean (SD)
**Outcome Variables**		
Physical Activity (Yes = 1) (*n* = 16,906)		21.2
Limited Screen Time (Yes = 1) (*n* = 16,988)		54.1
Adequate Sleep (Yes = 1) (*n* = 16,899)		65.9
**Child Characteristics**		
Sex (Male = 1) (*n* = 18,705)	9721	51.5
Age (*n* = 18,705)		11.6 (3.4)
Race/Ethnicity (*n* = 18,705)		
White, Non-Hispanic	14,784	55.9
Black, Non-Hispanic	1420	15.5
Hispanic	2501	28.6
Special Health Care Needs (Yes = 1) (*n* = 18,705)	5394	23.6
**Parental/Household Characteristics**		
Mother’s Age (*n* = 18,285)		29.0 (6.1)
Parental Educational Attainment (*n* = 18,705)		
Less Than High School	477	11.1
High School or Trade/Vocational School	2522	19.8
Some College/Associate Degree	4548	22.0
College Degree or Higher	11,158	47.2
Family Structure (*n* = 18,330)		
Two Parents, Married	12,858	62.1
Two Parents, Not Married	1155	7.8
Single Parent	3525	21.3
Other Family Types ^b^	792	5.8
Household Income ^c^ (*n* = 15,327)		
99% FPL or Below	1423	19.2
100–199% FPL	2205	21.8
200–399% FPL	4964	29.1
400% FPL or Higher	6735	29.9
Household Size (*n* = 18,705)		4.4 (1.3)
Government Assistance Program Participation (Yes = 1) (*n* = 18,705)	4657	39.2
**Neighborhood Characteristics**		
Supportive Neighborhood (Yes = 1) (*n* = 18,240)	11,096	55.1
Neighborhood Amenities (Yes = 1) (*n* = 18,705)	15,898	86.7
Distracting Neighborhood Conditions (Yes = 1) (*n* = 18,244)	4009	27.4

*Notes.* SD = Standard Deviation; FPL = Federal Poverty Level; ^a^ Total *n* for each variable may vary due to missing values; ^b^ Other family types include grandparent household and other relation, including foster care home; ^c^ Frequency of household income without imputed values is presented. Outcome definitions: Physical activity (=Yes) indicates 60 min of activity every day in the past week; Limited screen time (=Yes) indicates ≤2 h of recreational screen time on most weekdays; Adequate sleep (=Yes) indicates 9–11 h/night for ages 6–12 and 8–10 h/night for ages 13–17.

**Table 2:** Descriptive Statistics for Individual and Cumulative Adverse Childhood Experiences.

ACEs (Adverse Childhood Experiences)	Unweighted *n* ^a^	Weighted %
**Individual ACE (Yes = 1)**		
Hard to cover basics like food or housing (*n* = 18,363)	2438	16.4
Parent or guardian divorced or separated (*n* = 18,138)	5072	29.5
Parent or guardian died (*n* = 18,126)	700	4.1
Parent or guardian served time in jail (*n* = 18,084)	1373	9.1
Saw or heard parents or adults slap, hit, kick, or punch one another in the home (*n* = 18,052)	1086	6.9
Was a victim of violence or witnessed violence in the neighborhood (*n* = 18,065)	836	5.6
Lived with anyone who was mentally ill, suicidal, or severely depressed (*n* = 18,053)	1994	10.4
Lived with anyone who had a problem with alcohol or drugs (*n* = 18,066)	2097	11.0
Treated or judged unfairly because of his or her race or ethnic group (*n* = 18,119)	624	5.8
**Cumulative ACEs** (*n* = 18,705)		
0 ACEs	10,607	54.6
1 ACE	4227	22.6
2 ACEs	1798	10.0
3 or More ACEs	2073	12.8

*Notes.* ^a^ Total *n* for each variable may vary due to missing values.

**Table 3:** Results of Multivariate Logistic Regression Models Predicting Physical Activity, Limited Screen Time, and Adequate Sleep among Children Aged 6–17 Years.

	Physical Activity (*n* = 16,906)		Limited Screen Time (*n* = 16,988)		Adequate Sleep (*n* = 16,899)	
	AOR	95% CI	AOR	95% CI	AOR	95% CI
**Child Characteristics**						
Child Sex (Male = 1)	1.38 ***	[1.17, 1.62]	0.82 **	[0.71, 0.95]	1.01	[0.87, 1.18]
Child Age	0.89 ***	[0.87, 0.91]	0.85 ***	[0.84, 0.87]	0.99	[0.97, 1.01]
Race/Ethnicity (Reference = White, Non-Hispanic)						
Black, Non-Hispanic	0.72 *	[0.55, 0.92]	0.76 *	[0.60, 0.96]	0.53 ***	[0.43, 0.66]
Hispanic	0.63 ***	[0.49, 0.82]	0.81 *	[0.67, 1.00]	0.86	[0.70, 1.06]
Special Health Care Needs (Yes = 1)	0.75 **	[0.63, 0.90]	0.76 ***	[0.65, 0.88]	0.93	[0.80, 1.08]
**Parental/Household Characteristics**						
Mother’s Age	1.00	[0.98, 1.01]	0.98 *	[0.97, 1.00]	0.99	[0.98, 1.01]
Family Structure (Reference = Two Parents, Married)						
Two Parents, Not Married	0.81	[0.58, 1.13]	0.93	[0.69, 1.25]	0.73	[0.53, 1.01]
Single Parent	0.93	[0.71, 1.23]	0.93	[0.73, 1.17]	0.87	[0.68, 1.11]
Other Family Types	1.30	[0.84, 2.00]	0.95	[0.63, 1.43]	1.12	[0.76, 1.66]
Parental Educational Attainment (Reference = College Degree or Higher)						
Less Than High School	1.30	[0.83, 2.02]	0.83	[0.54, 1.27]	0.61 *	[0.40, 0.92]
High School or Trade/Vocational School	1.22	[0.93, 1.62]	0.86	[0.69, 1.07]	0.65 ***	[0.52, 0.82]
Some College/Associate Degree	1.34 *	[1.09, 1.64]	0.94	[0.79, 1.11]	0.86	[0.72, 1.04]
Household Income (Reference = 400% FPL or Higher)						
99% FPL or Below	1.23	[0.89, 1.70]	1.32	[0.95, 1.84]	0.92	[0.69, 1.24]
100–199% FPL	1.01	[0.76, 1.36]	1.04	[0.81, 1.33]	0.82	[0.65, 1.04]
200–399% FPL	0.89	[0.72, 1.09]	0.94	[0.79, 1.12]	0.80 *	[0.67, 0.96]
Household Size	0.96	[0.89, 1.04]	0.99	[0.92, 1.07]	0.96	[0.89, 1.03]
Government Assistance Program Participation (Yes = 1)	1.08	[0.82, 1.42]	0.73 **	[0.59, 0.91]	0.88	[0.71, 1.09]
**Neighborhood Characteristics**						
Supportive Neighborhood (Yes = 1)	1.29 **	[1.08, 1.53]	1.33 ***	[1.14, 1.56]	1.04	[0.89, 1.23]
Neighborhood Amenities (Yes = 1)	0.85	[0.69, 1.04]	1.05	[0.85, 1.30]	1.06	[0.87, 1.31]
Distracting Neighborhood Conditions (Yes = 1)	0.95	[0.78, 1.17]	0.98	[0.81, 1.18]	1.04	[0.86, 1.25]
**Adverse Childhood Experiences (Reference = 0 ACEs)**						
1 ACE	0.88	[0.71, 1.08]	0.74 **	[0.61, 0.89]	0.82	[0.67, 1.00]
2 ACEs	0.99	[0.72, 1.36]	0.70 *	[0.53, 0.92]	0.70 *	[0.52, 0.92]
3 or More ACEs	1.21	[0.85, 1.71]	0.69 *	[0.51, 0.93]	0.67 **	[0.50, 0.89]

*Notes.* AOR = Adjusted Odds Ratio; CI = Confidence Interval; FPL = Federal Poverty Level; * *p* < 0.05; ** *p* < 0.01; *** *p* < 0.001. Outcome definitions: Physical activity (=Yes) indicates 60 min of activity every day in the past week; Limited screen time (=Yes) indicates ≤2 h of recreational screen time on most weekdays; Adequate sleep (=Yes) indicates 9–11 h/night for ages 6–12 and 8–10 h/night for ages 13–17.

**Table 4:** Interaction Effects of ACEs with Child Sex and Race/Ethnicity on Physical Activity, Limited Screen Time, and Adequate Sleep.

	Physical Activity (*n* = 16,906)		Limited Screen Time (*n* = 16,988)		Adequate Sleep (*n* = 16,899)	
	AOR	95% CI	AOR	95% CI	AOR	95% CI
**Child Sex**						
Female	Reference		Reference		Reference	
Male × 1 ACE	0.76	[0.51, 1.13]	0.92	[0.65, 1.30]	0.86	[0.60, 1.24]
Male × 2 ACEs	0.61	[0.36, 1.05]	0.93	[0.57, 1.52]	1.09	[0.67, 1.77]
Male × 3 or More ACEs	1.08	[0.62, 1.87]	1.11	[0.67, 1.84]	1.22	[0.76, 1.97]
**Race/Ethnicity**						
White, Non-Hispanic	Reference		Reference		Reference	
Black, Non-Hispanic × 1 ACE	1.78 *	[1.02, 3.10]	1.24	[0.76, 2.04]	1.17	[0.72, 1.90]
Black, Non-Hispanic × 2 ACEs	1.81	[0.92, 3.55]	0.67	[0.37, 1.21]	0.68	[0.38, 1.21]
Black, Non-Hispanic × 3 or More ACEs	1.25	[0.61, 2.56]	1.48	[0.70, 3.10]	1.47	[0.79, 2.75]
Hispanic × 1 ACE	1.20	[0.65, 2.23]	0.86	[0.52, 1.42]	0.93	[0.56, 1.55]
Hispanic × 2 ACEs	1.44	[0.64, 3.26]	0.87	[0.44, 1.73]	0.46 *	[0.24, 0.87]
Hispanic × 3 or More ACEs	1.87	[0.89, 3.92]	1.42	[0.75, 2.70]	0.86	[0.46, 1.61]
**Model Interaction *p*-value**						
ACEs × Child Sex	0.20	0.91	0.58	0.20	0.91	0.58
ACEs × Race/Ethnicity	0.21	0.28	0.12	0.21	0.28	0.12

*Notes.* AOR = Adjusted Odds Ratio; CI = Confidence Interval. Reference category = 0 ACEs. The results are adjusted for covariates at the individual, parent, household, and neighborhood levels. * *p* < 0.05. Outcome definitions: Physical activity (=Yes) indicates 60 min of activity every day in the past week; Limited screen time (=Yes) indicates ≤2 h of recreational screen time on most weekdays; Adequate sleep (=Yes) indicates 9–11 h/night for ages 6–12 and 8–10 h/night for ages 13–17.

## Data Availability

The data analyzed in this study are publicly available from the United States Census Bureau website. Specifically, the data were obtained from the National Survey of Children’s Health, available at: https://www.census.gov/programs-surveys/nsch.html (accessed on 20 November 2020).
